# Supplementary material for: Production, Characterization, and Assessment of Permanently Cationic and Ionizable Lipid Nanoparticles for Use in the Delivery of Self-Amplifying RNA Vaccines
Source: Pharmaceutics. 2023 Apr 7;15(4):1173. doi: 10.3390/pharmaceutics15041173 (PMC10143526; doi:10.3390/pharmaceutics15041173)
Supplement: Supplementary file 1 [file pharmaceutics-15-01173-s001.zip › pharmaceutics-2301426-supplementary.pdf]

SUPPLEMENTARY INFORMATION

Production, characterization, and assessment of permanently cationic and ionizable Lipid Nanoparticles for use in the delivery of self-amplifying RNA vaccines

Dylan Kairuz[1], Nazia Samudh[1], Abdullah Ely[1], Patrick Arbuthnot[1], Kristie Bloom[1*]

1 Wits/SAMRC Antiviral Gene Therapy Research Unit, Faculty of Health Sciences, Infectious Diseases and Oncology Research Institute (IDORI), University of the Witwatersrand, Johannesburg, South Africa

* Correspondence: Kristie.Bloom@wits.ac.za; Tel.: +27 (0)11 717 1206

Supplementary Methods

Gel retardation assay:

To assess RNA complexation efficacy of saRNA-Ext-LNPs, a gel retardation assay was performed. saRNA-Ext-cLNPs were produced and formulated with saRNA-eGFP at different N:P ratios. Samples were mixed with 2× RNA Loading Dye (New England Biolabs, Massachusetts, USA) containing ethidium bromide. These were run on a 1% denaturing formaldehyde agarose gel and imaged thereafter. Naked saRNA-eGFP was used as a negative control to identify uncomplexed or free saRNA in saRNA-Ext-cLNP samples.

Supplementary Figures

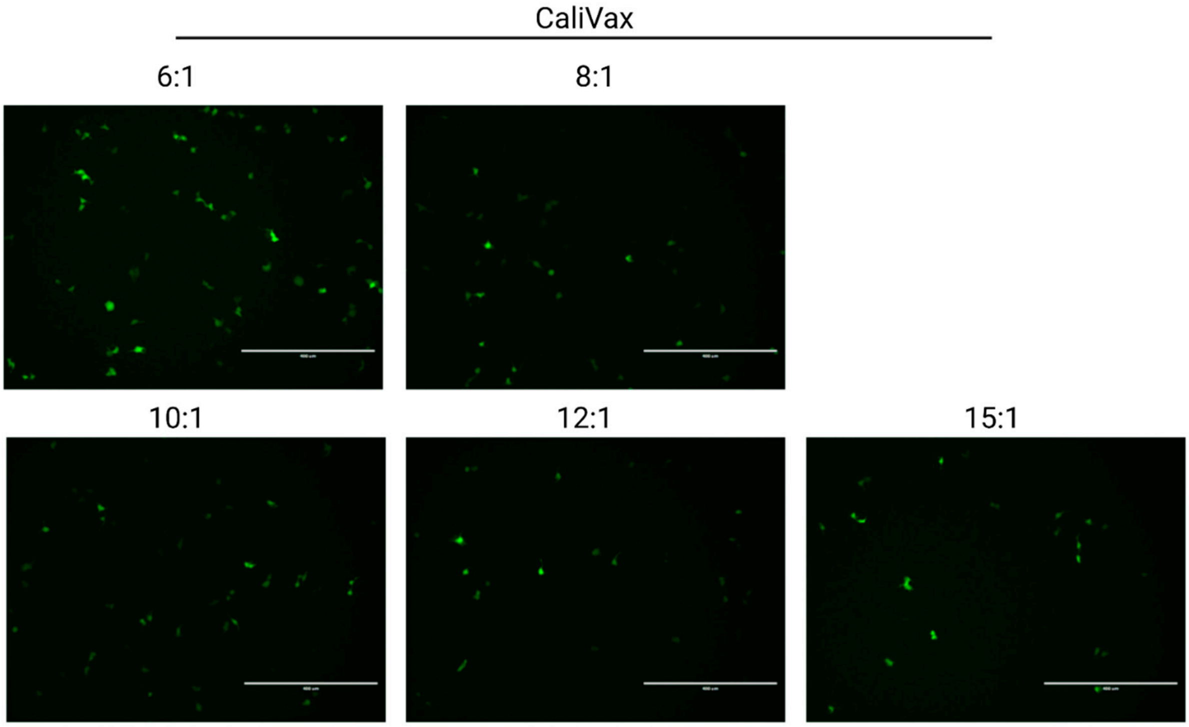

Figure S1. saRNA-Ext-CaliVax optimization. Various N:P ratios were tested to determine the optimal ratio for transfection of saRNA. Other than the optimal N:P ratio shown in Figure 4a, higher N:P ratios were found to be less efficient at delivering saRNA. This included 8:1, 10:1, 12:1 and 15:1 N:P ratios. Images were taken at 24 hours post-transfection. Created with BioRender.com

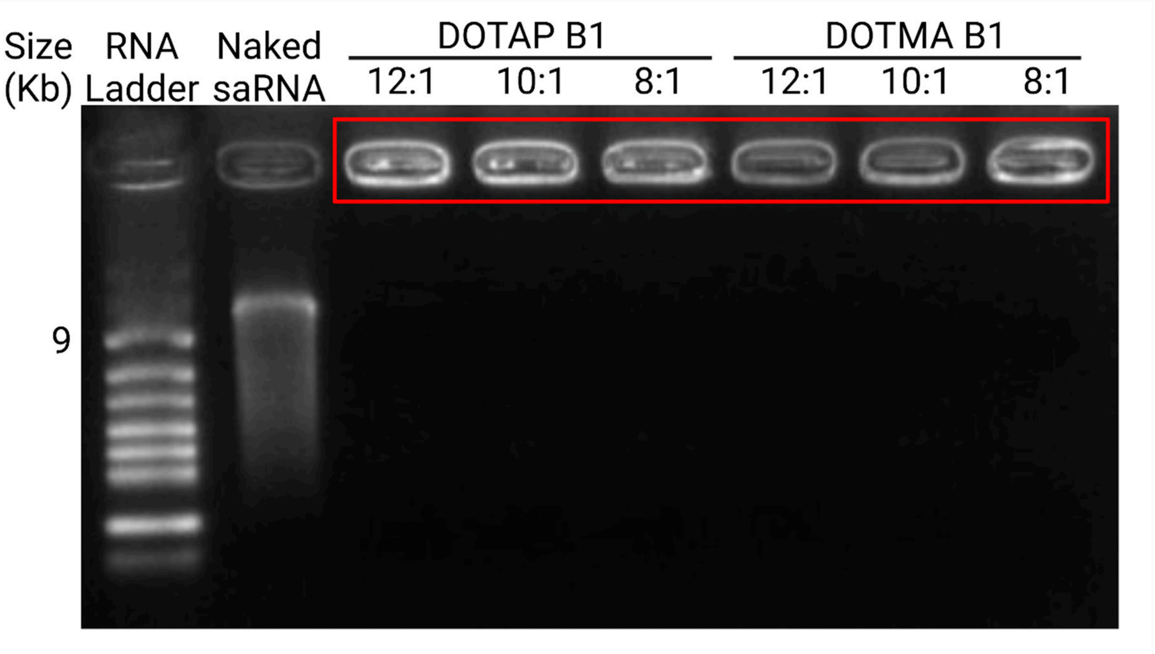

Figure S2. Gel retardation assay of DOTAP and DOTMA B1 saRNA-Ext-cLNPs. Retardation of the sample in the saRNA-LNP sample wells (outlined in red) is evident as compared to the naked saRNA control, where a distinct band can be visualized just above 9 kb. This shows efficient saRNA complexation to the exterior of the LNPs preventing free RNA migrating through the agarose gel. Created with BioRender.com

Supplementary Tables

Table S1 - Primers used for qRT-PCR of transcripts encoding innate immune response proteins

Gene	Amplicon size	Primer Name	Primer Sequence (5'-3')
OAS1	84	OAS1-F	CGA GGG AGC ATG AAA ACA CAT TT
		OAS1-R	GCA GAG TTG CTG GTA GTT TAT GAC
PKR	171	PKR-F	GAAGTGGACCTCTACGCTTTGG
		PKR-R	TGATGCCATCCCGTAGGTCTGT
IFN-β	110	IFN-β-F	TCC AAA TTG CTC TCC TGT TGT GCT
		IFN-β-R	CCA CAG GAG CTT CTG ACA CTG AAA A
IFIT1	182	IFIT-F	CCC TGA AGC TTC AGG ATG AAG G
		IFIT-R	AGA AGT GGG TGT TTC CTG CAA G
GAPDH	226	GAPDH-F	GAA GGT GAA GGT CGG AGT C
		GAPDH-R	GAA GAT GGT GAT GGG ATT TC

Table S2 - Delta Ct, fold change and statistics for qRT-PCR.

Mock	1000 g		500 µg		250 µg	
	ΔCt (Mean)	SEM	ΔCt (Mean)	SEM	ΔCt (Mean)	SEM
IFNβ	18.34	0.33	16.00	0.11	16.20	0.22
IFIT	9.57	0.16	9.43	0.03	9.22	0.10
OAS	22.04	0.28	23.42	0.27	22.43	0.22
PKR	7.23	0.29	7.24	0.07	7.37	0.11

CCAU-saRNA-Luc2	1000 g				500 µg				250 µg			
	ΔCt (Mean)	SEM	Fold change	P-value	ΔCt (Mean)	SEM	Fold change	P-value	ΔCt (Mean)	SEM	Fold change	P-value
IFNβ	15.84	0.23	5.52	0.0048	17.26	0.27	0.43	0.0297	16.14	0.08	1.03	0.8072
IFIT	8.28	0.29	2.50	0.0296	9.24	0.21	1.17	0.4634	9.12	0.10	1.06	0.5692
OAS	18.35	0.51	13.81	0.0069	20.89	0.32	5.83	0.0040	21.50	0.14	1.88	0.0323
PKR	7.05	0.24	1.12	0.6601	7.20	0.19	1.04	0.8639	7.20	0.11	1.12	0.3507

Poly I:C	1000 g				500 µg				250 µg			
	ΔCt (Mean)	SEM	Fold change	P-value	ΔCt (Mean)	SEM	Fold change	P-value	ΔCt (Mean)	SEM	Fold change	P-value
IFNβ	12.69	0.39	51.44	0.0004	15.42	0.18	1.50	0.0655	16.44	0.08	0.83	0.3946
IFIT	4.66	0.35	31.46	0.0015	7.69	0.21	3.41	0.0128	9.05	0.08	1.12	0.2748
OAS	12.81	0.49	647.60	0.0004	15.75	0.35	208.63	0.00009	21.71	0.37	1.72	0.1907
PKR	6.63	0.18	1.48	0.1702	6.84	0.18	1.33	0.1468	7.05	0.06	1.24	0.0865

Note: Significant increases in mRNA expression are highlighted in orange.

Table S3 - Lipid compositions of formulated cLNPs

Formulation Code	Lipid composition	Lipid Molar Ratio
B1	Cationic Lipid:Chol:DOPE	35:49:16
F1	Cationic Lipid:Chol:DOPE	50:40:10
F2	Cationic Lipid:Chol:DSPC	50:40:10
G1	Cationic Lipid:Chol:DOPE	45:25:30
I1	Cationic Lipid:DOPE	50:50

Table S4 - saRNA-cLNP average sizes and PDIs as measured by dynamic light scattering

Type of saRNA Formulation (Ext/Int)	Formulation Code	N:P Ratio	Average Size (nm)	PDI
External	CaliVax	2.5:1	347.5 ± 17.3	0.42370 ± 0.040
	DOTAP F1	8:1	211.2 ± 4.84	0.3101 ± 0.037
	DOTMA F1	12:1	162.2 ± 1.5	0.1946 ± 0.019
	DOTAP G1	8:1	167.8 ± 1.525	0.1162 ± 0.025
	DOTMA G1	8:1	174.30 ± 1.72	0.08463 ± 0.013
	DDA B1	8:1	294.1 ± 4.97	0.3631 ± 0.025
	DOTAP B1	8:1	167.3 ± 1.15	0.1679 ± 0.033
	DOTMA B1	12:1	174 ± 1.19	0.1408 ± 0.0052
	DOTAP F2	4:1	574.6 ± 1.41	0.371 ± 0.05
	DOTMA F2	4:1	235.9 ± 22.64	0.41 ± 0.026
	DOTMA I1	8:1	185.3 ± 0.4474	0.1745 ± 0.028
	DOTAP I1	8:1	171.8 ± 2.443	0.2079 ± 0.032
Internal	DOTAP F1	15:1	160.8 ± 1.51	0.3025 ± 0.021
	DOTAP F1	12:1	187.4 ± 3.51	0.3691 ± 0.009
	DOTAP F1	8:1	220.5 ± 8.49	0.436 ± 0.0798
	DDA F2	12:1	377.3 ± 6.912	0.4349 ± 0.029
	DOTMA F1	12:1	287.8 ± 5.177	0.4527 ± 0.005
	DOTAP B1	12:1	166.4 ± 0.1455	0.2555 ± 0.03
	DOTAP B1	8:1	151.2 ± 1.62	0.2597 ± 0.0058
	DOTMA B1	12:1	204.3 ± 3.39	0.293 ± 0.028
